# Toward interventions to reduce internalized ageism

**DOI:** 10.1093/geroni/igab046.2296

**Published:** 2021-12-17

**Authors:** Andrew Steward

**Affiliations:** University of Denver, Lone Tree, Colorado, United States

## Abstract

Ageism is an insidious form of injustice that is internalized from an early age with accumulating negative health impacts across the lifespan. Internalized ageism is associated with numerous public health outcomes, including physical and mental health, functional impairment, cognition, cardiovascular stress, hospitalizations, and longevity. Research has begun to document how ageism negatively impacts health through psychological, behavioral, and physiological pathways. Yet, limited research has addressed interventions to reduce internalized ageism. This study integrates stereotype embodiment theory, theories of successful and productive aging, and recent scholarly literature to present a conceptual model with potential downstream, midstream, and upstream interventions at micro, meso, and macro levels. Micro interventions include: social, physical, and cognitive engagement, as well as stress management. Meso interventions include: education, intergenerational contact, and narrative reframing. Macro interventions include anti-ageism policy, such as amendments to the Age Discrimination in Employment Act (ADEA). The conceptual model is described in detail, and implications for practitioners are discussed. The need to examine how policy influences health through the three pathways in stereotype embodiment theory is discussed. This study provides a working model for scholars and practitioners to use when considering paths toward reducing internalized ageism and optimizing well-being for aging adults.

